# Remnant Lipoprotein Cholesterol and Cardiovascular and Cerebrovascular Events in Patients with Non-Alcoholic Fatty Liver Disease

**DOI:** 10.3390/jcm7110378

**Published:** 2018-10-23

**Authors:** Daniele Pastori, Francesco Baratta, Marta Novo, Nicholas Cocomello, Francesco Violi, Francesco Angelico, Maria Del Ben

**Affiliations:** 1 I Clinica Medica, Department of Internal Medicine and Medical Specialties, Sapienza University of Rome, Viale del Policlinico 155, 00161 Rome, Italy; francesco.baratta@uniroma1.it (F.B.); marta.novo@uniroma1.it (M.N.); nicholas.cocomello@gmail.com (N.C.); francesco.violi@uniroma1.it (F.V.); maria.delben@uniroma1.it (M.D.B.); 2 Department of Anatomical, Histological, Forensic Medicine and Orthopedics Sciences, Sapienza University of Rome, 00161 Rome, Italy; 3 Department of Public Health and Infectious Diseases, Sapienza University of Rome, Viale del Policlinico 155, 00161 Rome, Italy

**Keywords:** NAFLD, cardiovascular events, cholesterol remnants

## Abstract

Non-alcoholic fatty liver disease (NAFLD) is characterized by an atherogenic dyslipidaemia and an increased cardiovascular risk. Remnant lipoprotein cholesterol (RLP-C) is emerging as a novel cardiovascular risk factor, but its predictive value in patients with NAFLD is unknown. We investigated factors affecting RLP-C levels, and the association with major adverse cardiovascular and cerebrovascular events (MACCE) in NAFLD. A prospective observational cohort study was carried out including 798 unselected patients with cardio-metabolic diseases screened by ultrasound for the presence of NAFLD. Fasting RLP-C (mg/dL) was calculated as total cholesterol—(HDL (high-density lipoprotein) + LDL (low-density-lipoprotein)). Primary endpoint of the follow-up study was a combined endpoint of MACCE. Patients with NAFLD (79.2%) had higher median fasting RLP-C in comparison to those without (27.0 vs. 20.0 mg/dL, respectively *p* < 0.001). Metabolic syndrome, NAFLD, age above median, and female sex were independently associated to fasting RLP-C above the median. In patients with NAFLD, values of RLP-C were associated with liver disease severity, as shown by the increasing value of RLP-C across tertiles of aspartate aminotransferase (AST) (*p* = 0.002) and gamma-glutamyl transpeptidase (GGT) (*p* < 0.001). Furthermore, levels of RLP-C and Hamaguchi score, were significantly correlated (*r* = 0.193, *p* < 0.001). During a median follow-up of 32 months (interquartile range: 14.2–51.7, 1700 person-years), 41 MACCE (2.41%/year) were registered in 596 NAFLD patients. The rate of events was higher in NAFLD patients with RLP-C above the median compared to those below (log-rank test *p* = 0.040). Age (hazard ratio (HR) 1.039, 95% confidence interval (CI), 1.005–1.074, *p* = 0.024), previous cardiovascular events (HR 2.210, 95% CI, 1.052–4.643, *p* = 0.036), female sex (HR 0.454, 95% CI, 0.208–0.989, *p* = 0.047) and RLP-C above the median (HR 2.202, 95% CI, 1.132–4.285, *p* = 0.020) were associated with MACCE. In conclusion, we found that NAFLD was independently associated with higher circulating RLP-C, and that high RLP-C levels were predictive of MACCE in patients with NAFLD.

## 1. Introduction

Non-alcoholic fatty liver disease (NAFLD) is the most common liver disease worldwide and will soon represent the first cause of liver transplantation in the near future [1]. In addition to liver-related complications, people with NAFLD have also an increased chance of developing cardiovascular diseases (CVD), such as myocardial infarction and stroke, which represent the major causes of death in this setting [2]. This risk is mainly attributable to the several cardio-metabolic risk factors frequently associated with NAFLD, mainly represented by metabolic syndrome (MetS) features [3]. Furthermore, patients with NAFLD have been shown to have additional risk factors contributing to cardiovascular risk such as, endothelial dysfunction, imbalance of oxidative status and cardiac abnormalities [4,5,6,7].

A typical dyslipidaemia characterized by increased serum triglycerides, low high-density lipoprotein (HDL) cholesterol and increased small, dense low-density-lipoproteins (LDL) particles, i.e., the so-called ‘atherogenic lipid triad’ has been frequently described in patients with NAFLD [8,9,10]. In the Multi-Ethnic Study of Atherosclerosis (MESA) study, lipoprotein abnormalities were significantly associated with the severity of NAFLD [11]. In particular the odds ratio of having an atherogenic dyslipidaemia increased from 1.62 for mild NAFLD, to 3.17 for severe NAFLD [11].

Among lipoprotein fractions, remnant lipoprotein cholesterol (RLP-C) plays a key role in atherosclerosis [12]. In the fasting condition, RLP-C is the cholesterol contained in very-low-density-lipoproteins (VLDL), which originate in the liver, and in their relatively TG-depleted remnants, which are known as “triglyceride-rich lipoproteins”.

Of note, RLP-C is emerging as a risk factor for CVD; however, only a few studies have investigated the predictive value of RLP-C against CVD, and none of them has been performed in patients with NAFLD [13,14,15,16,17].

The aims of the study were to investigate (1) the association between NAFLD and RLP-C levels in a large cohort of patients with cardio-metabolic risk factors screened for the presence of liver steatosis and (2) the relationship between fasting RLP-C and major adverse cardiovascular and cerebrovascular events (MACCE) incidence in patients with NAFLD.

## 2. Materials and Methods

The study was carried out in 811 consecutive outpatients referring to the Day Service of Internal Medicine and Metabolic Disorders of the Policlinico Umberto I University Hospital in Rome with at least one out of the following cardio-metabolic diseases: arterial hypertension, overweight/obesity, type 2 diabetes, dyslipidaemia, atrial fibrillation (AF), MetS (Adult treatment panel III criteria). Patients were screened with ultrasound (US) for the presence of NAFLD with the exclusion of those who matched the following exclusion criteria: average daily consumption of alcohol >20 g in women and of >30 g in men (assessed by Alcohol Use Disorders Identification Test, AUDIT); presence of hepatitis B surface antigen and antibody to hepatitis C virus; positive tests for autoimmune hepatitis; cirrhosis and other chronic liver diseases; diagnosis of oncological diseases and concomitant therapy with drugs known to promote liver steatosis (e.g., amiodarone); other chronic infectious or autoimmune disease.

Patients underwent routine laboratory analyses in fasting condition, including blood glucose, and liver function tests. Serum total cholesterol, HDL and LDL cholesterol and triglycerides were measured by an Olympus AN 560 apparatus using an enzymatic colorimetric method. Fasting RLP-C was calculated as (total cholesterol—(HDL + LDL)) and expressed as mg/dL. Aspartate aminotransferase(AST) to Platelet Ratio Index (APRI) was also calculated.

Liver US scanning was performed to assess the presence of steatosis in all patients. All US were performed by the same operator who was blinded to laboratory values using a GE Vivid S6 apparatus equipped with a convex 3.5 MHz probe. Liver steatosis was defined according to Hamaguchi criteria [18].

### 2.1. Cardiovascular Endpoints

The occurrence of MACCE was considered as the primary endpoint of the follow-up study. MACCE included fatal/nonfatal ischemic stroke and myocardial infarction (MI), cardiac (stent or coronary artery bypass surgery/CABG) or peripheral revascularization (carotid endarterectomy or lower limb percutaneous transluminal angioplasty, PTA), new-onset supraventricular arrhythmias (such as AF), and cardiovascular death. Diagnosis of MI was made according to the third universal definition of myocardial infarction [19]. Ischemic stroke was determined on clinical manifestations and confirmed by radiological findings. If a patient died within 4 weeks of MI or stroke, this event was recorded as fatal MI/stroke. Transient ischemic attack (TIA) was defined according to the Classification of Cerebrovascular Diseases III [20].

Death was classified as cardiovascular unless there was an unequivocal non-cardiovascular cause of death. Cardiovascular death included sudden death; progressive congestive heart failure; procedure-related death. Only the first MACCE event registered during follow-up was used in the analysis.

#### Statistical Analysis

Distribution of variables was assessed by the Kolmogorov-Smirnov test. Categorical variables were reported as percentages, continuous variables as means +/− standard deviation or medians and interquartile ranges (IQR) depending on their distribution. Independence of categorical variables was tested by *χ*^2^ test. Student unpaired *t* and Mann-Whitney *U* tests were used to compare means and medians, respectively. Group comparisons were performed by the Kruskal-Wallis test and the Spearman rank correlation test was used for bivariate correlation analysis.

Continuous variables with non-normal distribution were log-transformed for multivariable analysis and dichotomized when possible. Multivariable logistic regression analysis was used to calculate the odds ratio (OR) for predictors of fasting RLP-C above median in the whole cohort and in the subgroup of patients with NAFLD. After dividing the NAFLD population into two groups according to the median value of RLP-C, the cumulative incidence of MACCE was estimated using a Kaplan-Meier product-limit estimator, and survival curves were formally compared using the log-rank test. Cox’s proportional hazards regression analysis was used to calculate the adjusted relative hazard ratios (HR) of MACCE by each clinical variable.

Variables with a *p* < 0.05 at univariate analysis were included in the multivariable models. Only *p* values <0.05 were considered as statistically significant. All tests were two-tailed and analyses were performed using computer software packages (SPSS-18.0, SPSS Inc., Chicago, IL, USA).

The study protocol was approved by the local ethical board of Sapienza—University of Rome (Ref. 2277 prot. 873/11)—and was carried out according to the principles of the Declaration of Helsinki. All patients provided signed written informed consent at entry.

## 3. Results

### 3.1. Factors Associated to Levels of RLP-C above Median

Out of the 811 patients, 13 refused to be enrolled and 798 were included in the analysis. Table 1 reports the clinical and biochemical characteristics of patients according to the median value of RLP-C. Patients with RLP-C above median were younger, less frequently women, and with higher serum total cholesterol, triglycerides, LDL-cholesterol and lower HDL-cholesterol. Moreover, they also had higher serum ALT, AST, GGT, and blood glucose values (Table 1).

Prevalence of MetS and NAFLD was significantly higher in patients with fasting RLP-C above median as compared to those below median (Table 1).

At multivariate logistic regression analysis MetS, NAFLD, were positively associated to fasting RLP-C above median, while age above median and female sex was negatively associated (Table 2, Model A).

### 3.2. Subgroup of Patients with NAFLD

**Figure 1:**
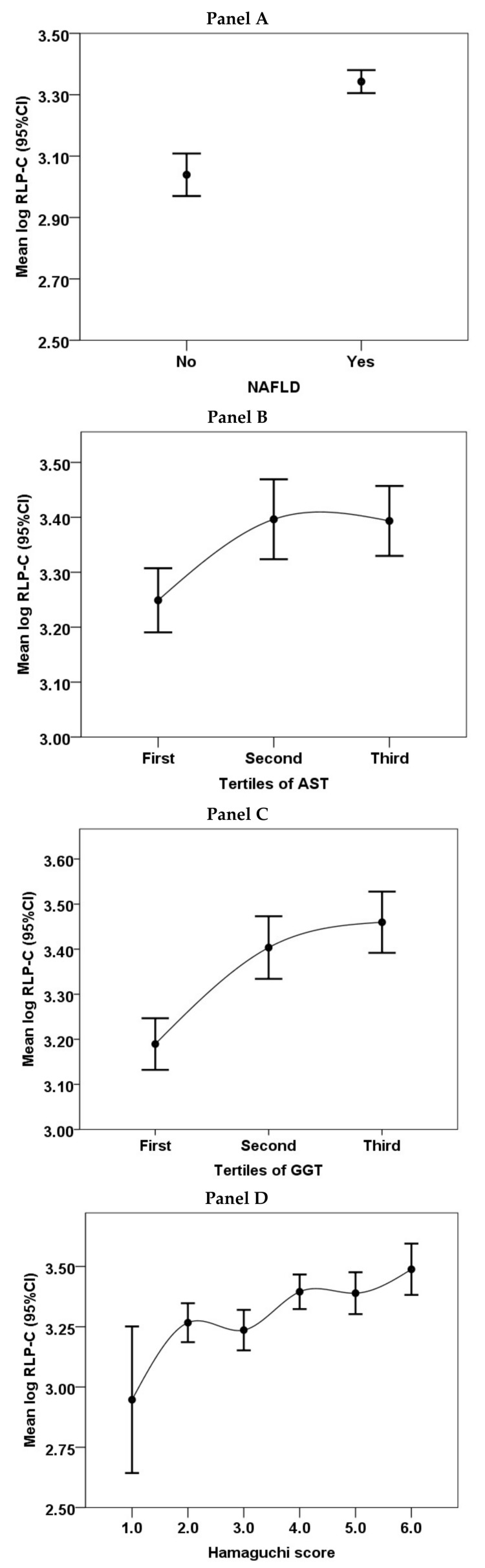
Values of remnant lipoprotein cholesterol (RLP-C) in patients with and without non-alcoholic fatty liver disease (NAFLD) (**Panel A**) and across tertiles of Aspartate aminotransferase (AST) (**Panel B**) and Gamma-glutamyl transpeptidase (GGT) (**Panel C**) and according to the Hamaguchi score (**Panel D**) in patients with NAFLD.

Patients with NAFLD had higher median fasting RLP-C in comparison to those without (27.0 (IQR 20.0–37.3) vs. 20.0 (IQR 15.5–26.0) mg/dL, respectively *p* < 0.001 (Figure 1, Panel A).

In patients with NAFLD, values of RLP-C increased across tertiles of AST (*p* = 0.002, Figure 1 Panel B) and GGT (*p* < 0.001, Figure 1 Panel C), and Hamaguchi score (*p* < 0.001, Figure 1 Panel D). Values of RLP-C were significantly correlated to the Hamaguchi score (*r* = 0.193, *p* < 0.001).

A multivariable logistic regression analysis showed that in patients with NAFLD, age above median and female sex were inversely associated with RLP-C above median, while MetS was directly associated (Table 2, Model B).

Median APRI was 0.2 (IQR 0.2–0.3), and 25 (3.96%) patients had APRI >0.7. Having RLP-C above median increased the chance of having a value of APRI above median (univariable OR 1.51, 95%CI 1.07–2.13, *p* = 0.017), also after adjustment for confounders listed in Table 2 model B (multivariable OR 1.49, 95%CI 1.01–2.21, *p* = 0.045).

### 3.3. Predictors of MACCE 

Of the 632 patients with NAFLD, 36 (5.7%) were lost at follow-up. Thus, 596 were included in the survival analysis. Median follow-up time was 32 months (IQR: 14.2–51.7) yielding 1700 person-years of observation. At follow-up, 41 NAFLD patients (2.41%/year) experienced a MACCE: 17 MI/cardiac revascularizations (1%/year), 4 TIAs, 3 ischaemic strokes, 10 PTAs, 4 new-onset AF, 3 cardiovascular deaths.

**Figure 2:**
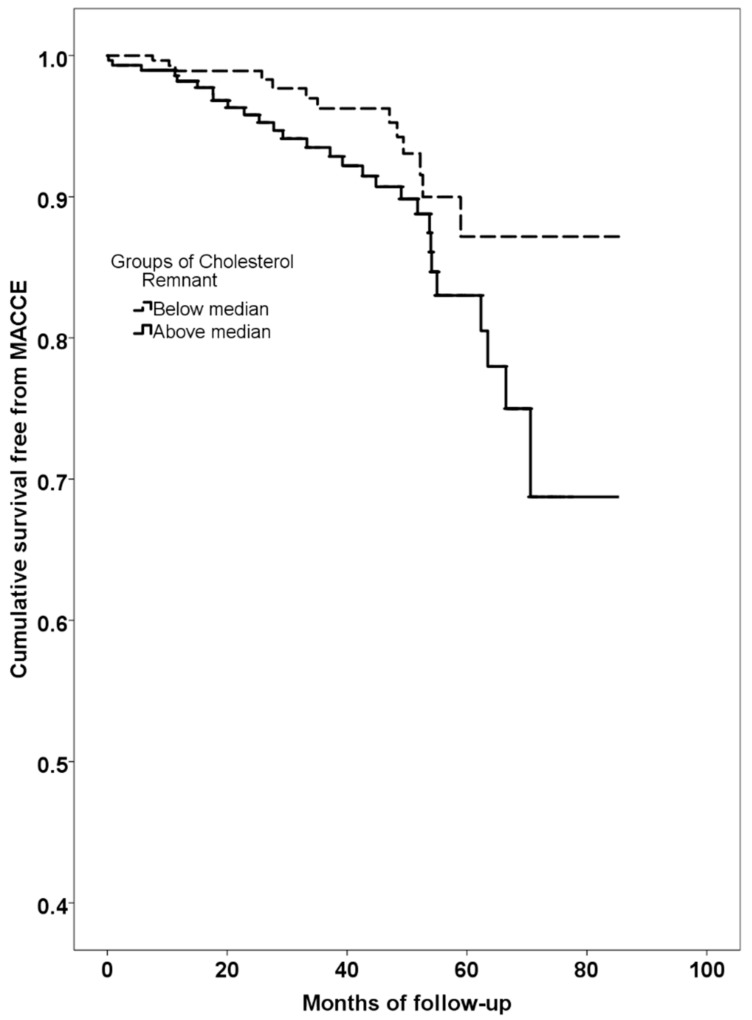
Kaplan–Meier curves estimate of survival free from major cardiovascular and cerebrovascular events (MACCE) according to groups of cholesterol remnant value (Dotted line: below median. Continuous line: above median).

A higher rate of MACCE was found in NAFLD patients with RLP-C above median compared to those below (log-rank test *p* = 0.040, Figure 2).

Univariate Cox proportional hazard analysis showed that age, previous cardiovascular events, MetS and RLP-C (above median) were associated with an increased risk of MACCE, while women disclosed a lower risk (Table 3).

On multivariable Cox proportional hazard analysis, age, previous cardiovascular events, and RLP-C above the median were positively associated with MACCE, while female sex showed a negative association (Table 3).

## 4. Discussion

In this prospective observational study, we found that NAFLD was independently associated with higher serum fasting RLP-C, and that high RLP-C levels were predictive of MACCE in patients with NAFLD.

In this cohort of consecutive patients with cardio-metabolic disorders screened by ultrasound for the presence of liver steatosis, we found that metabolic syndrome, NAFLD, age above median and female sex were independently associated with fasting RLP-C above median.

In particular, in patients with NAFLD, values of RLP-C were associated with liver disease severity, as shown by the increasing value of RLP-C across tertiles of AST and GGT. Furthermore, we found a significant correlation between levels of RLP-C and Hamaguchi score, suggesting that cholesterol remnants may contribute to the severity of fatty liver accumulation in this patients’ population.

Another interesting finding of the present study relies on the association between RLP-C and the risk of fibrosis. Thus, when we divided the cohort of patients with NAFLD according to the median value of APRI, which is a validated non-invasive marker of liver fibrosis, we found that patients with high RLP-C were more likely to have an APRI index above the median. Altogether these results suggest that RLP-C may play a role in liver disease severity.

When we analyzed the association between high RLP-C levels and MACCE incidence in patients with NAFLD, we found that the annual rate of MACCE in our cohort was 2.47% confirming a high cardiovascular risk in this population [21]. Thus, this incidence is higher than that observed in the general Italian population, in whom the incidence rate of coronary heart disease is 0.64/100 person-years in men and 0.16/100 person-years in women [22]. We found that increasing age, high levels of RLP-C and previous cardiovascular events were significant predictors of MACCE in our cohort.

Previous studies investigated the predictive role of RLP-C against cardiovascular events in different clinical settings. However, most of the studies performed so far measured RLP-C by immunoseparation technique using Apo-AI and Apo-B antibodies [13,14,17,23,24], and some others measured non-fasting RLP-C [25], which includes also cholesterol of chylomicrons of intestinal origin and their remnants. For all these reasons, a direct comparison of the results is difficult, despite almost all of these studies found an increased risk of cardiovascular events with increasing RLP-C concentration. A large report on the United States population including the Jackson Heart and Framingham Offspring Cohort Studies measured RLP-C as the sum of VLDL and intermediate-density lipoprotein (IDL) and found similar circulating RLP-C levels observed in our study (~30 mg/dL) [26]. The authors found that high RLP-C levels were predictive of incident coronary disease in primary prevention subjects [26].

We also found a lower risk of MACCE in women. This finding is in keeping with previous data showing a relatively lower risk of cardiovascular events for women in the pre- and peri-menopausal period as compared to middle-age men [27]. However, the specific contribution of gender on cardiovascular risk in patients with NAFLD has been poorly investigated. In a previous study we found that women with NAFLD had a better endothelial function, as assessed by the non-invasive flow-mediated dilation, which may partially be responsible for the lower risk of MACCE in women [5]. In addition, a previous study including 309 patients with NAFLD, showed an increased estimated 10-year risk of cardiovascular events for men as compared to women [28]. Larger prospective studies are needed to further explore this issue. Moreover, the relationship between post-menopausal status and cardiovascular risk in relation to the presence of NAFLD has not been investigated so far.

Our study has clinical implications. We explored for the first time the association between NAFLD and RLP-C, and its role in determining cardiovascular risk. Our findings suggest RLP-C as a previously unrecognized risk factor for CVD in NAFLD. This finding is of concern in patients with NAFLD, as increased fasting RLP-C, together with hypertriglyceridemia, low HDL-cholesterol and small and dense LDL, may play a causal role in the development of premature atherosclerosis in patients with NAFLD. In fact, RLP-Cs may cross the vascular endothelial wall carrying into the artery wall up to 20 times as much cholesterol per particle as LDL. Then, they are taken by resident macrophages promoting the formation of foam cells [29].

This is of particular interest as RLP-C has been shown to be associated to incident CVD in patients already taking statins [16], and also in patients who reached an appropriate LDL cholesterol target (<100 mg/dL) [15]. Furthermore, not all statins seem to have the same effect on RLP-C, with simvastatin and atorvastatin being more effective than pravastatin in lowering RLP-C levels [30]. The relationship between RLP-C and statin treatment in NAFLD deserves more research, as statins have been shown to be safe and effective for the treatment of NAFLD [31,32] especially in patients with increased liver enzymes [33], but are frequently not prescribed because of feared potential side effects [34].

A further still unexplored issue is the predictive value of fasting RLP-C on the progression of fatty liver disease, which needs to be evaluated in longitudinal studies.

This study has limitations that should be mentioned. The observational design is an intrinsic limitation of the present study, which does not allow a cause-effect relationship between RLP-C and the other variables of interest to be found. In particular, the use of a combined endpoint does not allow the association between RLP-C and specific types of cardiovascular events to be seen. Finally, patients were recruited from a single hospital-based center.

In conclusion, our results show an independent association between NAFLD and increased serum levels of RLP-C. High RLP-C was associated with an increased risk of MACCE in NAFLD patients, suggesting a role for fasting RLP-C in increasing cardiovascular risk in this setting.

## Figures and Tables

**Table 1:** Characteristics of patients with fasting remnant lipoprotein cholesterol (RLP-C) below and above median.

	Whole Population (*n* = 798)	Patients with RLP-C below Median (<25.5 mg/dL) (*n* = 399)	Patients with RLP-C above Median (≥25.5 mg/dL) (*n* = 399)	*p*
**Age (years)**	56.6 ± 12.8	58.1 ± 13.1	55.2 ± 12.4	0.002
**Women** **(%)**	37.3	41.4	33.3	0.023
**Body mass index (kg/m^2^)**	29.6 ± 5.0	29.1 ± 5.1	30.1 ± 4.9	0.004
**Waist circumference (cm)**	105.1 ± 12.2	103.6 ± 12.7	106.5 ± 11.7	0.001
**Total cholesterol (mg/dL)**	198.1 ± 41.1	188.4 ± 37.5	207.7 ± 42.4	<0.001
**High density lipoprotein cholesterol (mg/dL)**	49.5 ± 14.0	54.6 ± 14.7	44.5 ± 11.4	<0.001
**Low density lipoprotein cholesterol (mg/dL)**	118.8 ± 36.2	115.2 ± 33.4	122.4 ± 38.5	0.006
**Triglycerides (mg/dL)**	150.8 ± 101.2	95.7 ± 29.5	206.2 ± 116.4	<0.001
**Alanine aminotransferase (IU/L)**	24.0 (17.0–39.0)	22.0 (16.0–34.0)	27.0 (20.0–42.0)	<0.001
**Aspartate aminotransferase (IU/L)**	21.0 (17.0–27.0)	20.0 (16.0–25.0)	22.0 (18.0–29)	<0.001
**Gamma-glutamyl transpeptidase (IU/L)**	25.0 (17.0–40.0)	21.0 (16.0–33.0)	28.5 (19.0–46.0)	<0.001
**Platelets (10^9^/L)**	236.1 ± 60.4	233.8 ± 58.3	238.3 ± 62.3	0.321
**Blood Glucose (mg/dL)**	103.8 ± 27.9	100.5 ± 21.1	107.0 ± 32.9	0.001
**Glycated haemoglobin (HbA1c) (%)**	5.8 ± 1.1	5.7 ± 1.0	5.9 ± 1.1	0.007
**Type 2 diabetes (%)**	26.6	24.1	29.1	0.109
**Arterial hypertension (%)**	61.1	62.1	60.2	0.610
**Systolic blood pressure (mmHg)**	130 (120–140)	130 (120–140)	130 (120–140)	0.384
**Diastolic blood pressure (mmHg)**	80 (75–85)	80 (70–85)	80 (75–85)	0.226
Previous cardiovascular vascular disease (%) *	13.0	15.2	10.8	0.072
**Metabolic Syndrome (%)**	52.0	31.5	72.0	<0.001
**Non-alcoholic fatty liver disease (%)**	79.2	69.2	89.2	<0.001
**Statin therapy (%)**	40.8	37.7	43.8	0.090

* Including previous cardiovascular and cerebrovascular events and baseline atrial fibrillation.

**Table 2:** Multivariable logistic regression analysis of factors associated with fasting remnant lipoprotein cholesterol (above median) in the whole population (Model A) and in patients with non-alcoholic fatty liver disease (NAFLD) (Model B).

Model A. Whole Cohort	Adjusted Odds Ratio (95% Confidence Intervals)	*p*
**Age above median**	0.494 (0.348–0.701)	<0.001
**Female sex**	0.671 (0.482–0.936)	0.019
**Previous cardiovascular events**	0.794 (0.476–1.324)	0.376
**Non-alcoholic fatty liver disease**	2.246 (1.480–3.411)	<0.001
**Metabolic Syndrome**	5.933 (4.219–8.344)	<0.001
**Model B. NAFLD patients**		
**Age above median**	0.550 (0.374–0.808)	0.002
**Female sex**	0.684 (0.472–0.990)	0.044
**Previous cardiovascular events**	0.791 (0.443–1.414)	0.430
**Metabolic Syndrome**	6.588 (4.534–9.573)	<0.001

**Table 3:** Univariable and multivariable Cox proportional hazard regression analysis of factors associated with major cardiovascular and cerebrovascular events (MACCE) in patients with NAFLD.

Univariable	*p* Value	Hazard Ratio	95% Confidence Interval	
**Age**	0.001	1.050	1.019	1.081
**Female sex**	0.031	0.428	0.198	0.926
**Previous cardiovascular events**	<0.001	3.442	1.802	6.573
**Arterial hypertension**	0.113	1.785	0.872	3.653
**Diabetes**	0.586	1.192	0.634	2.239
**Body mass index**	0.898	0.996	0.939	1.057
**Waist circumference**	0.269	1.014	0.990	1.038
Metabolic syndrome *	0.042	2.233	1.030	4.840
**Cholesterol remnant (above median)**	0.044	1.966	1.018	3.800
**Multivariable**	***p*** ** Value**	**Hazard Ratio**	**95% Confidence Interval**	
**Age**	0.024	1.039	1.005	1.074
**Female sex**	0.047	0.454	0.208	0.989
**Cholesterol remnant (above median)**	0.020	2.202	1.132	4.285
**Previous cardiovascular events**	0.036	2.210	1.052	4.643

* Metabolic syndrome was not used in the multivariable Cox proportional hazard ratio analysis as it includes dyslipidemia.
